# Usefulness of DNA Obtained from FFPE Tissue Sections Stained with Masson’s Trichrome in Forensic Identification: A Pilot Study

**DOI:** 10.3390/genes16121416

**Published:** 2025-11-28

**Authors:** María-de-Lourdes Chávez-Briones, Adriana Ancer-Arellano, Ivett Miranda-Maldonado, Juan M. Solís-Soto, Jaime García-Juárez, Marta Ortega-Martínez, Gilberto Jaramillo-Rangel

**Affiliations:** 1 Department of Pathology, School of Medicine, Autonomous University of Nuevo Leon, Monterrey 64460, Mexico; mdlourdes.chavezbrn@uanl.edu.mx (M.-d.-L.C.-B.); adar7035@gmail.com (A.A.-A.); ivettmiranda77@gmail.com (I.M.-M.); marta.ortegamrt@uanl.edu.mx (M.O.-M.); 2 Department of Physiology, School of Dentistry, Autonomous University of Nuevo Leon, Monterrey 64460, Mexico; solis.sotojm@gmail.com; 3 Department of Histology, School of Medicine, Autonomous University of Nuevo Leon, Monterrey 64460, Mexico; jaime.garciajr@uanl.edu.mx

**Keywords:** formalin-fixed and paraffin-embedded (FFPE) tissues, forensic identification, Masson’s trichrome staining

## Abstract

Background/Objectives: Formalin-fixed paraffin-embedded (FFPE) tissues are sometimes the only DNA source for forensic applications. The quantity and integrity of the DNA extracted from these samples depend on multiple factors. In this work, we analyzed, for the first time, whether Masson’s trichrome (MT) staining alters the results of genetic profiles obtained from DNA extracted from FFPE tissue sections. Methods: Three pairs of sections from the year 2024 and three pairs from the year 2001 were analyzed. Each pair consisted of serial sections, one stained with hematoxylin and eosin and the other with MT. DNA was extracted using the PrepFiler Express BTA™ Forensic DNA Extraction Kit and quantified by real-time PCR using the Quantifiler™ HP DNA Quantification Kit. DNA samples were processed for short tandem repeat (STR) profiling using the GlobalFiler™ PCR Amplification Kit. The amplified alleles were separated and analyzed using an ABI PRISM^®^ 3500 genetic analyzer. Results: All MT-stained samples showed deficiency in most or all of the parameters assessed: DNA yield, degradation index, number of alleles detected, random match probability value, and intensity of the electropherogram peaks. In fact, DNA could not even be quantified in the samples processed in 2001. Conclusions: These results could be due to the large number of acids used in MT staining, which cause chemical modification and hydrolysis of DNA, affecting the success of PCR-based methods used subsequently. In conclusion, DNA obtained from MT-stained FFPE tissue sections may be highly degraded and should therefore be used with great caution in forensic settings.

## 1. Introduction

Forensic laboratories use PCR-based genotyping of short tandem repeats (STRs) as the standard DNA analysis method for applications such as paternity testing and identification of individuals. Formalin-fixed paraffin-embedded (FFPE) tissues are sometimes the only DNA source for forensic applications. However, this DNA is often scarce and degraded, and FFPE samples contain remnants of xylene, which inhibits the proteinase K used in the DNA extraction procedure, and of formalin, which inhibits the PCR reaction [1,2]. On the other hand, there is controversy about the extent to which the storage time of FFPE samples is related to the integrity and quantity of the DNA obtained, and whether it could significantly affect PCR amplification [3,4].

FFPE tissue sections are stained to enhance their microscopic visualization in examination. The most common staining method in human histology and histopathology is hematoxylin and eosin (H&E) technique. There have been conflicting reports as to whether H&E staining has negative effects on DNA molecular assays [5]. However, studies that have systematically investigated the influence of H&E staining on STR profiling using multiplex systems (as is done in forensic identification procedures) have not found such staining to have a significant effect on the results, both in cell smears [6,7] and in FFPE samples [8,9].

Another staining method useful in histopathological analysis is Masson’s trichrome (MT) technique. As its name suggests, this technique uses three dyes to selectively stain muscle fibers red (Biebrich scarlet), collagen fibers blue or green (depending on whether aniline blue or light green is used), and cell nuclei black (Weigert’s iron hematoxylin) [10]. MT staining is used in some laboratories as a routine stain for kidney and liver biopsies, and is a crucial technique in histopathology for analyzing the relative proportion of tissue components [11,12]. It can be useful in the study of connective tissue diseases by evaluating conditions such as fibrosis, scarring, and tissue remodeling, and to differentiate between collagen and smooth muscle in tumors [13,14,15]. In addition, it can be used in the analysis of diseases such as nonalcoholic fatty liver disease, hepatitis, cardiomyopathy, myocardial infarction, and idiopathic pulmonary fibrosis [16]. It has also been used to identify protozoa in tissue sections from the gastrointestinal tract [17,18]. To the best of our knowledge, no studies have yet been performed to determine whether MT staining alters the results of STR profiles obtained from FFPE tissue sections.

In this work, differences between STR profiles derived from archived FFPE tissue sections stained with H&E or MT were analyzed to determine whether the latter staining could influence the results obtained in forensic identification processes.

## 2. Materials and Methods

**Figure 1:**
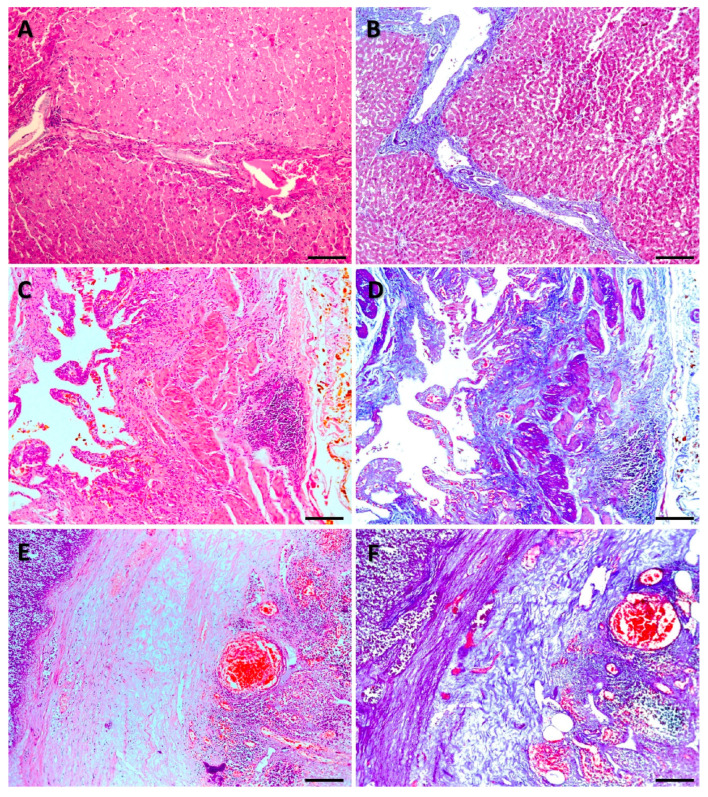
Representative microphotographs of the tissues analyzed. Samples processed in 2024 and stained with hematoxylin and eosin (H&E) or Masson’s trichrome (MT). Liver focal nodular hyperplasia stained with H&E (**A**) and MT (**B**); Acute cholecystitis with lithiasis stained with H&E (**C**) and MT (**D**); Acute appendicitis with peritonitis stained with H&E (**E**) and MT (**F**). Scale bar 100 µm.

**Figure 2:**
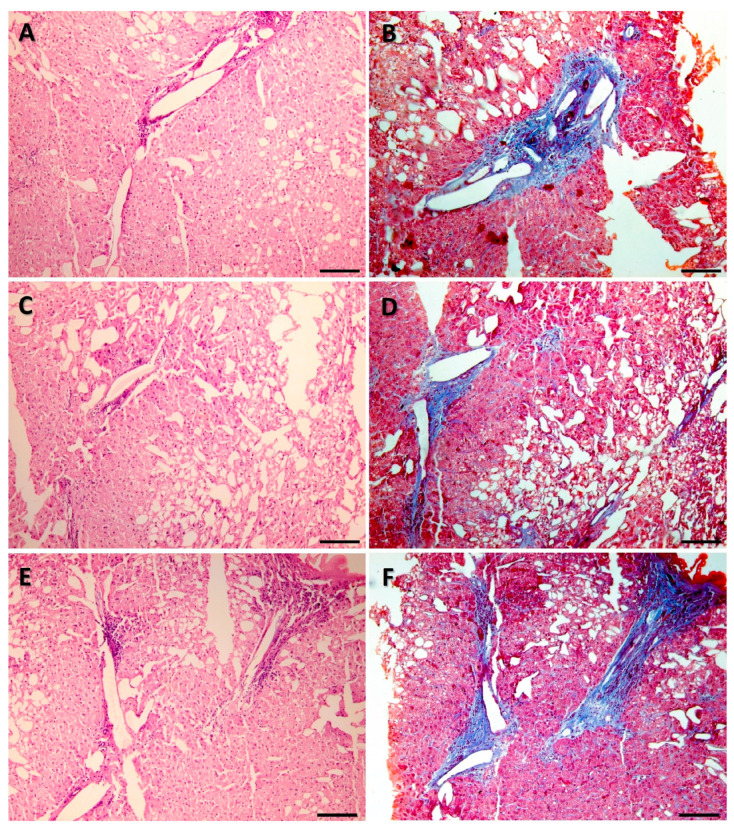
Representative microphotographs of the tissues analyzed. Samples processed in 2001. The three pairs of samples were of macro- and microvesicular steatosis stained with hematoxylin and eosin (**A**,**C**,**E**) and Masson’s trichrome (**B**,**D**,**F**). Scale bar 100 µm.

FFPE tissue sections from the archives of the Department of Pathology, School of Medicine, Autonomous University of Nuevo León, located in Monterrey, Mexico, were analyzed. Three pairs of sections from the year 2024 and three pairs of sections from the year 2001 were selected. Each pair consisted of serial sections, one stained with H&E and the other stained with MT. The diagnoses of the analyzed samples are summarized in Table 1. All samples were 5 microns thick and processed for paraffin embedding, sectioning, staining with H&E or MT, and mounting with standard histopathology procedures [10]. Representative microscopic images of the samples analyzed are shown in Figure 1 and Figure 2.

The coverslips were removed from the microscopic slides by soaking overnight in xylene at 56 °C. Sections were deparaffinized in xylene and hydrated in a decreasing ethanol series and distilled water. Tissues were scraped from slides with sterile scalpels and collected in microcentrifuge tubes containing 1 mL distilled water.

DNA extraction from the tissues was performed using the PrepFiler Express BTA™ Forensic DNA Extraction Kit (Applied Biosystems, Foster City, CA, USA). Lysis buffer included in the kit was added to the samples together with Proteinase K (200 μg/mL) and 1 M dithiothreitol (DTT). Samples were incubated at 56 °C overnight under constant agitation. After centrifugation, the supernatants were subjected to DNA extraction using the AutoMate™ Express Forensic DNA Extraction System (Applied Biosystems, Foster City, CA, USA) according to the manufacturer’s protocol.

DNA was quantified by real-time PCR using the Quantifiler™ HP DNA quantification kit on the 7500 ABI detection system following the manufacturer’s recommendations (Applied Biosystems, Foster City, CA, USA). This quantification kit includes an internal PCR control (IPC) to detect the presence of PCR inhibitors, and can determine the degradation index (DI) by dividing the concentration of a small amplicon (80 bp) by that of a large amplicon (214 bp). Data analysis was performed using HID Real-Time PCR Analysis Software v1.3 (Applied Biosystems, Foster City, CA, USA). According to the manufacturer, for results to be considered reliable, the slope of the standard curve should be in the range of −3.0 to −3.6 for the small amplicon and −3.1 to −3.7 for the large amplicon, and the correlation coefficient (R^2^) value should be equal to or greater than 0.99 for both curves. Also, PCR inhibition was assumed in a sample when its IPC cycle threshold (CT) showed a shift ≥ 2 cycles compared to the CT value of the non-template control (NTC).

DNA samples (1 ng) were processed for STR profiling using the GlobalFiler™ PCR Amplification Kit and the ProFlex™ PCR Thermal Cycler according to the manufacturer’s recommended protocols (Applied Biosystems, Foster City, CA, USA). Amplified PCR products were electrophoretically separated on an ABI PRISM^®^ 3500 genetic analyzer as recommended by the manufacturer (Applied Biosystems, Foster City, CA, USA). GeneMapper^®^ IDX-v1.6 software (Applied Biosystems, Foster City, CA, USA) was used for genotyping. An analytical threshold of 55 relative fluorescence units (RFU) was used for allele labeling, with a stochastic threshold of 300 RFU for designation of homozygotes.

The random match probability (RMP) values were calculated using STR allele frequency data from our population (Mexican mestizo) and PATPCR software version 2.0.2 [19,20].

## 3. Results

The DNA concentrations, the DIs, and the CTs of the small and large amplicons obtained from the samples are shown in Table 2. The slope of the standard curve for the small amplicon was −3.067, and the R^2^ was 0.992. For the large amplicon, the slope of the standard curve was −3.317, and the R^2^ was 0.995. The IPC CT values of all samples were within ± 1 of the IPC CT of the NTC (CT = 29).

A higher amount of DNA was obtained from all sections processed in 2024 and stained with H&E than from their serial sections stained with MT. Regarding the samples processed in 2001, DNA was only obtained from those stained with H&E, while no DNA was obtained from those stained with MT.

All recent samples stained with H&E showed higher DNA concentrations than older samples stained with the same technique. Similarly, DNA was only obtained from recent samples stained with MT, while no DNA was obtained from older samples stained with MT.

DI values could only be obtained from all recent samples stained with H&E and from one recent sample stained with MT; DI values could not be obtained from all old samples and two recent samples stained with MT due to the failed amplification of the large target.

Table 3 shows the comparison between the STR profiles obtained from the samples processed in 2024, as well as the number of alleles recovered from each of them and their RMP values. A higher number of alleles and lower RMP values (higher discriminatory power) were obtained from H&E-stained sections compared to their respective MT-stained serial sections. Table 4 shows only the profiles obtained from the samples stained with H&E processed in 2001, since no DNA was obtained from the samples stained with MT from that year (Table 2). A lower number of alleles were obtained from all samples stained with H&E of 2001 compared to samples with the same staining processed in 2024.

**Figure 3:**
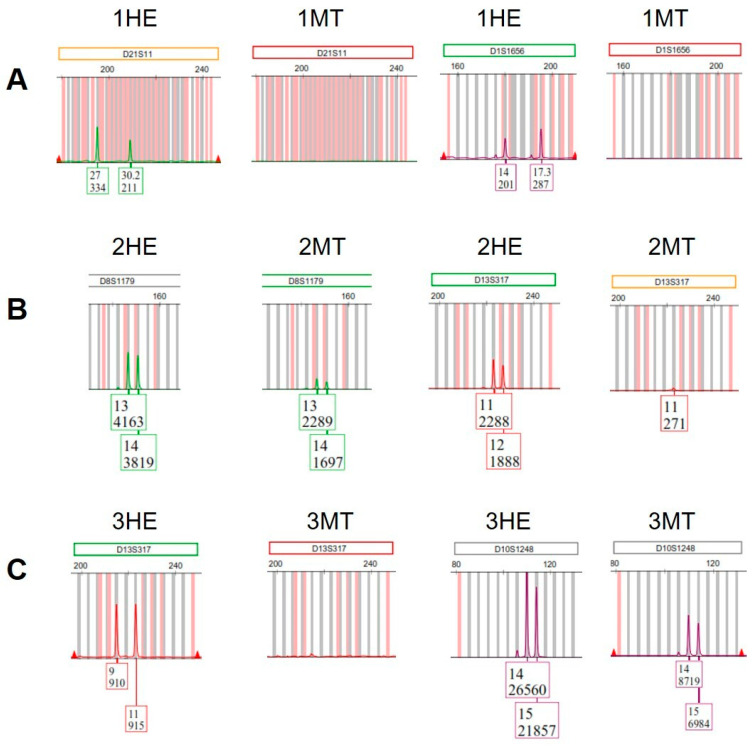
Fragments of electropherograms with examples of peaks obtained from samples processed in 2024, stained with hematoxylin and eosin (1-, 2-, and 3HE) or Masson’s trichrome (1-, 2-, and 3MT) (Supplementary Materials). (**A**) Sample 1MT had complete loss of both alleles at loci D21S11 and D1S1656; (**B**) At locus D8S1179, sample 2MT showed peaks with lower relative fluorescence units (RFU) values than those observed in sample 2HE. In addition, sample 2MT showed complete loss of one allele at the locus D13S317, while the other allele of this locus was observed with a lower RFU value than that of sample 2HE; (**C**) Sample 3MT had complete loss of both alleles at locus D13S317, while at locus D10S1248 showed peaks with lower RFU values than those observed in sample 3HE.

Figure 3 shows fragments of electropherograms with examples of peaks obtained from the samples processed in 2024. All peaks from the sections stained with MT (when were present) showed lower RFU values than their serial sections stained with H&E (Supplementary Materials).

## 4. Discussion

In forensic genetics laboratories, in the absence of samples known to contain sufficient quantity and quality of DNA, the need occasionally arises to obtain STR profiles from FFPE samples to serve as a reference for the identification of human remains or in paternity testing. The integrity and quantity of DNA extracted from these samples depend on multiple factors. In this work, we analyzed, for the first time, whether MT staining alters the results of STR profiles obtained from DNA extracted from FFPE tissue sections.

In this study, we included samples that were histopathologically processed recently and more than 20 years ago, with the goal of emulating situations that might arise in the actual practice of forensic analysis, that is, the resolution of new and old cases. Considering the results as a whole, samples stained with MT yielded results that were, in general, much inferior to those obtained with H&E-stained sections. In fact, in the samples processed in 2001 and stained with MT, not even DNA usable in STR profiling could be quantified.

Since the amount of DNA used for genotyping was the same for all samples (1 ng), the difference in the number of alleles obtained from each of them could depend on the presence or absence of PCR inhibitors. The IPC CT values of all samples were within ±1 of the IPC CT of the NTC. Thus, no evidence of inhibition was observed in any sample based on IPC CT delay ≥2. Therefore, the observed results could only be explained by the degree of DNA degradation, reflected in the DI of each sample.

Regarding the samples processed in 2024 and stained with H&E, detection of alleles at all loci analyzed was observed in 2HE and 3HE, while a partial profile was obtained for 1HE. These results were consistent with the sample DIs (2 and 11 for 2HE and 3HE, respectively, versus 86 for 1HE) (Table 2 and Table 3). Formalin fixation of tissues induces the development of several types of DNA damage, including deamination of cytosine bases, cross-linking between DNA molecules or between DNA and proteins, and DNA fragmentation, resulting in small DNA fragments that are not amenable for PCR amplification [21,22]. Although all three samples were processed in 2024, FFPE tissues have been reported to express varying degrees of DNA integrity even when processed in the same pathology laboratory and on similar dates. This is because the exact formalin concentration and pH can vary, and different fixation conditions can influence DNA quality and genotyping results [23,24].

Compared to the samples described above, the samples processed in 2001 and stained with H&E showed a lower DNA yield and a lower number of alleles, and it was not possible to calculate their DIs due to the failed amplification of the large target in the quantitative PCR, which indicated that their DNA was more degraded. Although there is controversy over the extent to which the storage time of FFPE tissues is related to a reduction in the ability to perform downstream molecular analyses, there is near consensus on one fact: DNA degrades over time. While this degradation could depend on several factors, such as the duration of fixation and the type of tissue [5,25,26], formalin fixation is considered to be the factor that contributes the most to it. Formaldehyde oxidizes to formic acid over time, gradually decreasing the pH during tissue storage and causing additional DNA degradation than described in the previous paragraph [27,28].

Although the RMP threshold that confers certainty that a genetic profile is unique in our population has not been calculated, this value is in the order of 1 × 10^−9^ to 1 × 10^−10^ in other populations [29,30]. In this work, the RMP values for the H&E-stained samples were between 5.12 × 10^−10^ and 7.53 × 10^−29^ (Table 3 and Table 4). Thus, despite the high degree of DNA degradation and/or the storage time of more than 20 years of some of those samples, their RMP values could be considered strong enough to assign, or at least confirm, an identity in a forensic identification process [29,30,31].

In contrast, only one of the MT-stained samples had an acceptable RMP value (sample 2MT, Table 3). In fact, all MT-stained samples showed deficiency in most or all of the parameters assessed, i.e., DNA yield, DI, number of alleles detected, RMP value, and intensity of the electropherogram peaks. All of these deficiencies arose from one common factor: the high degree of DNA degradation present in the samples.

In turn, this level of DNA degradation could be due to the large number of acids used in MT staining. Except for washings, MT-stained samples are subjected to the action of one or two acids at each step of the procedure. In total, five acids are used in this stain: picric, acetic, hydrochloric, phosphotungstic and phosphomolybdic (Table 5).

To the best of our knowledge, the effect of these acids, individually or collectively, on DNA obtained from MT-stained tissue sections has not been reported. However, the effect of picric, acetic, and hydrochloric acids on the yield and quality of DNA obtained in other contexts has been analyzed.

Bouin’s solution contains picric and acetic acid. Benerini Gatta et al. observed that DNA extracted from Bouin-fixed, paraffin-embedded samples is not suitable for PCR (among other molecular assays) due to its level of degradation [32]. Additionally, the acetic acid contained in some solutions used in fingerprint enhancement has been shown to decrease the amount of DNA recovered and the number of STR alleles obtained compared to reagents that do not contain the acid [33].

Decalcification agents containing hydrochloric acid have shown to cause a considerable decrease in both DNA yield and integrity, limiting further molecular analyses [34]. On the other hand, using a porcine model, Robino et al. evaluated DNA profiling of body parts treated with hydrochloric, nitric and sulfuric acid or aqua regia (nitric and hydrochloric acid in 1:3 volume ratio). Overall, the worst results were obtained with samples treated with hydrochloric acid and aqua regia [35].

At the molecular level, the main mechanism involved in the effect of acids on DNA integrity is the loss of bases (i.e., depurination and depyrimidination) through glycosidic bond cleavage. At the abasic sites formed, phosphodiester bonds become more susceptible to hydrolysis, resulting in DNA degradation [35,36].

Thus, apart from the factors already known to affect DNA integrity in FFPE tissue samples, in those stained with MT, the acids used in the staining procedure likely play a role. However, this hypothesis needs to be tested by analyzing a larger number of samples and conducting appropriate experiments. Strategies could be tested to mitigate this damage, for example, by replacing the wash water with Tris buffer pH 8.0, or with a 70% ethanol solution saturated with lithium carbonate, which has been shown to be effective in removing residual picric acid from Bouin-fixed tissues and improving DNA recovery [37].

A limitation of this work could be the small number of samples analyzed. However, the observed findings were consistent with each other and replicated in six different cases with samples processed on distant dates. On the other hand, analysis of a larger number of samples would help definitively establish whether MT-stained FFPE tissue sections can be used in paternity testing and identification of individuals. Also, investigations into the specific effects of the individual steps involved in MT staining could elucidate the causes of the findings observed in this study.

## 5. Conclusions

In conclusion, this is the first time the usefulness of DNA from MT-stained FFPE tissue sections for obtaining STR profiles has been reported. The results indicate that such DNA may be highly degraded and should therefore be used with great caution in forensic settings. The acids used in MT staining likely play a role in this degradation, causing chemical modification and hydrolysis of DNA, which affects the success of PCR-based methods used subsequently.

## Figures and Tables

**Table 1:** Characteristics of the analyzed samples.

Pathological Diagnosis	Year of Processing	Stain Technique	Sample ID
Liver focal nodular hyperplasia	2024	H&E	1HE
		MT	1MT
Acute cholecystitis with lithiasis	2024	H&E	2HE
		MT	2MT
Acute appendicitis with peritonitis	2024	H&E	3HE
		MT	3MT
Macro- and microvesicular steatosis	2001	H&E	4HE
		MT	4MT
Macro- and microvesicular steatosis	2001	H&E	5HE
		MT	5MT
Macro- and microvesicular steatosis	2001	H&E	6HE
		MT	6MT

H&E: hematoxylin and eosin; MT: Masson’s trichrome.

**Table 2:** Yield, degradation index and cycle thresholds of the small and large amplicons of DNA obtained from the FFPE tissue sections.

Sample	DNA Concentration (ng/µL)	Degradation Index	SA CT	LA CT
1HE	1.72	86	26	28
1MT	0.22	----	29	37
2HE	1.03	2	26	23
2MT	0.69	69	27	29
3HE	22.74	11	22	21
3MT	5.26	----	24	30
4HE	0.71	----	27	30
4MT	0.00	----	36	33
5HE	0.40	----	27	29
5MT	0.00	----	35	36
6HE	0.20	----	28	34
6MT	0.00	----	33	36

H&E: hematoxylin and eosin; MT: Masson’s trichrome; SA: small amplicon; LA: large amplicon; CT: cycle threshold; ----: no result was obtained.

**Table 3:** Comparison of short tandem repeats results of DNA recovered from FFPE tissue samples stained with hematoxylin and eosin (HE) or Masson’s trichrome (MT), processed in 2024.

Locus	1HE	1MT	2HE	2MT	3HE	3MT
D3S1358	15	15	16–18	16–18	15	15
vWA	16–18	-	16–17	16–17	14–18	-
D16S539	-	-	9–12	9	9–11	-
CSF1PO	-	-	11	-	11–13	-
TPOX	-	-	8–12	-	11	-
D8S1179	13–15	-	13–14	13–14	13–14	13–14
D21S11	27–30.2	-	31–31.2	31–31.2	28–30	-
D18S51	-	-	14–18	-	13	-
D2S441	10	10	10–11	10–11	11	11
D19S433	13.2–14	13.2–14	13.2–15.2	13.2–15.2	12–14	12–14
TH01	6–7	-	7	7	7–9.3	7–9.3
FGA	25–26	-	22–28	22–28	23–25	-
D22S1045	15	15	15–16	15–16	16–17	16–17
D5S818	11–12	-	11–12	11–12	11	11
D13S317	9–10	-	11–12	11	9–11	-
D7S820	-	-	11–12	-	8–9	-
SE33	-	-	27.2–28.2	27.2–28.2	18–26.2	-
D10S1248	13–14	13–14	15	15	14–15	14–15
D1S1656	14–17.3	-	16–17	16–17	14–18.3	-
D12S391	-	-	18–19	18–19	19–23	-
D2S1338	-	-	18–19	-	20	-
Amelogenin	XY	XY	XY	XY	XX	XX
Number of alleles detected	25	9	41	30	38	15
RMP	6.00 × 10^−16^	7.91 × 10^−5^	5.88 × 10^−27^	6.17 × 10^−22^	7.53 × 10^−29^	4.70 × 10^−8^

-: no result was obtained; RMP: random match probability.

**Table 4:** Short tandem repeats results of DNA recovered from FFPE tissue samples stained with hematoxylin and eosin, processed in 2001.

Locus	4HE	5HE	6HE
D3S1358	15–16	15–18	16–17
vWA	16–17	16–22	-
D16S539	-	-	-
CSF1PO	-	-	-
TPOX	-	-	-
D8S1179	13	12–13	12–13
D21S11	29	-	-
D18S51	-	-	-
D2S441	11–11.3	14–15	11–11.3
D19S433	14	12–14	14
TH01	9.3	9–9.3	-
FGA	21	21	-
D22S1045	15–16	15	11–17
D5S818	12–13	11–12	11
D13S317	11	-	-
D7S820	-	-	-
SE33	-	-	-
D10S1248	13–14	13–15	15–16
D1S1656	16	12–14	-
D12S391	19	21	-
D2S1338	-	-	-
Amelogenin	XY	XX	XY
Number of alleles detected	22	23	14
RMP	7.16 × 10^−17^	5.90 × 10^−19^	5.12 × 10^−10^

-: no result was obtained; RMP: random match probability.

**Table 5:** Acids contained in the reagents used in Masson’s trichrome staining.

Reagent	Acid(s)
Bouin’s solution	Picric acid Glacial acetic acid
Weigert’s iron hematoxylin	Hydrochloric acid
Biebrich scarlet	Glacial acetic acid
Differentiating/mordant solution	Phosphomolybdic acid Phosphotungstic acid
Aniline blue	Glacial acetic acid
Differentiating solution	Glacial acetic acid

## Data Availability

The original contributions presented in the study are included in the article, further inquiries can be directed to the corresponding author.

## Supplementary Materials

The following supporting information can be downloaded at https://www.mdpi.com/article/10.3390/genes16121416/s1. Figure S1: Electropherogram of sample 1HE; Figure S2: Electropherogram of sample 1MT; Figure S3: Electropherogram of sample 2HE; Figure S4: Electropherogram of sample 2MT; Figure S5: Electropherogram of sample 3HE; Figure S6: Electropherogram of sample 3MT.

## Abbreviations

The following abbreviations are used in this manuscript:

bp	Base pairs
°C	Degrees Celsius
CT	Cycle threshold
DI	Degradation index
DNA	Deoxyribonucleic acid
DTT	Dithiothreitol
FFPE	Formalin-fixed paraffin-embedded
H&E	Hematoxylin and eosin
IPC	Internal PCR control
µg	Micrograms
µL	Microliters
mL	Milliliters
MT	Masson’s trichrome
ng	Nanograms
NTC	Non-template control
pH	Negative logarithm of the hydrogen ion concentration
PCR	Polymerase chain reaction
R^2^	Correlation coefficient
RFU	Relative fluorescence units
RMP	Random match probability
STR	Short tandem repeat
