# Supplementary figures and images for: Usefulness of DNA Obtained from FFPE Tissue Sections Stained with Masson’s Trichrome in Forensic Identification: A Pilot Study
Source: Genes (Basel). 2025 Nov 28;16(12):1416. doi: 10.3390/genes16121416 (PMC12733228; doi:10.3390/genes16121416)

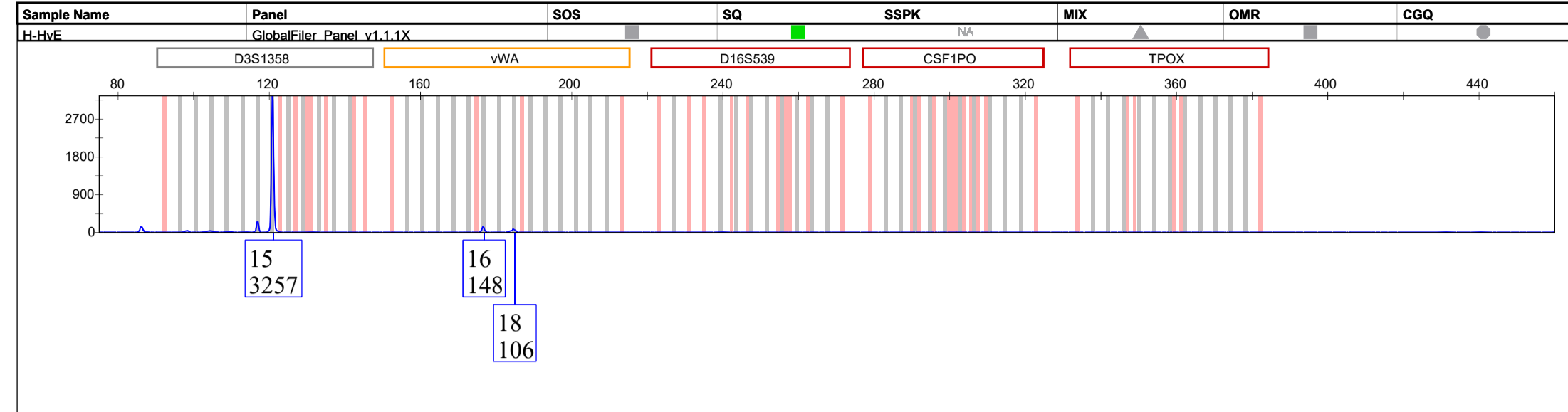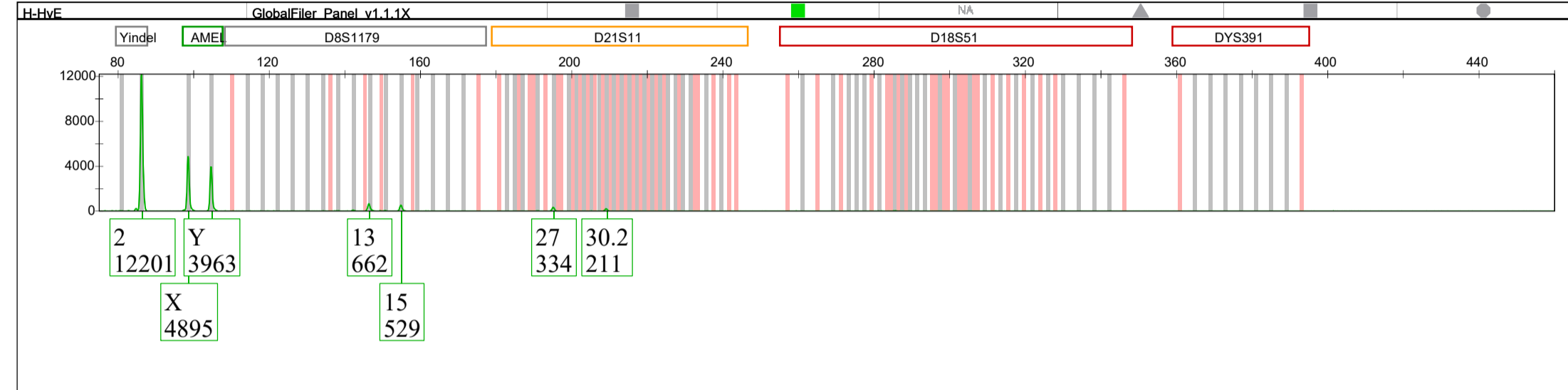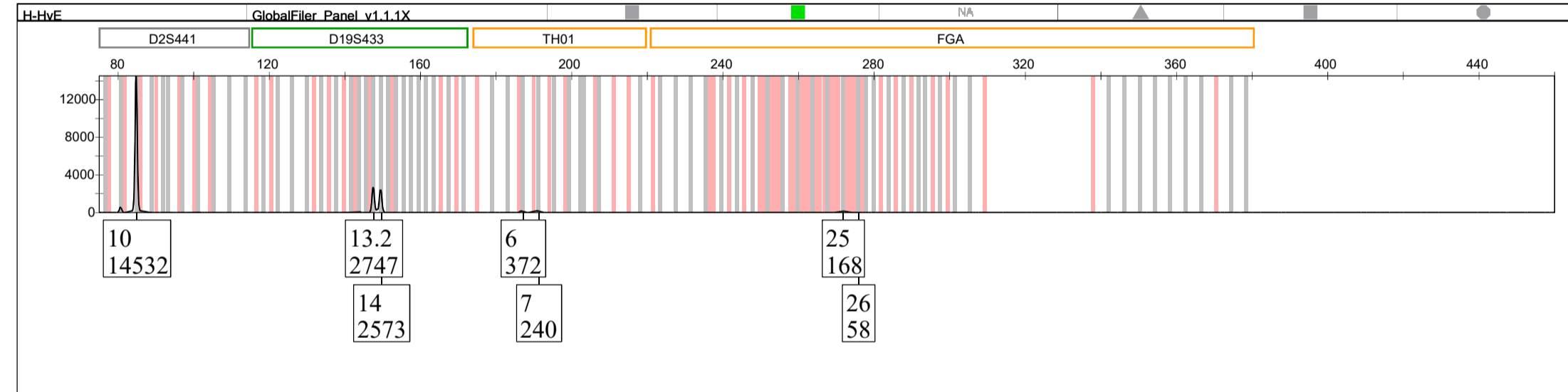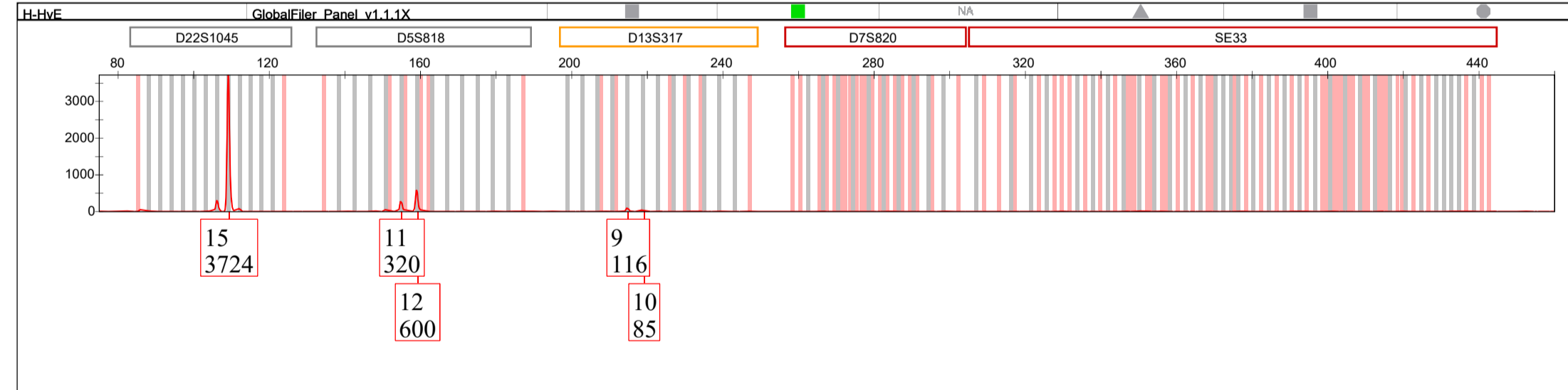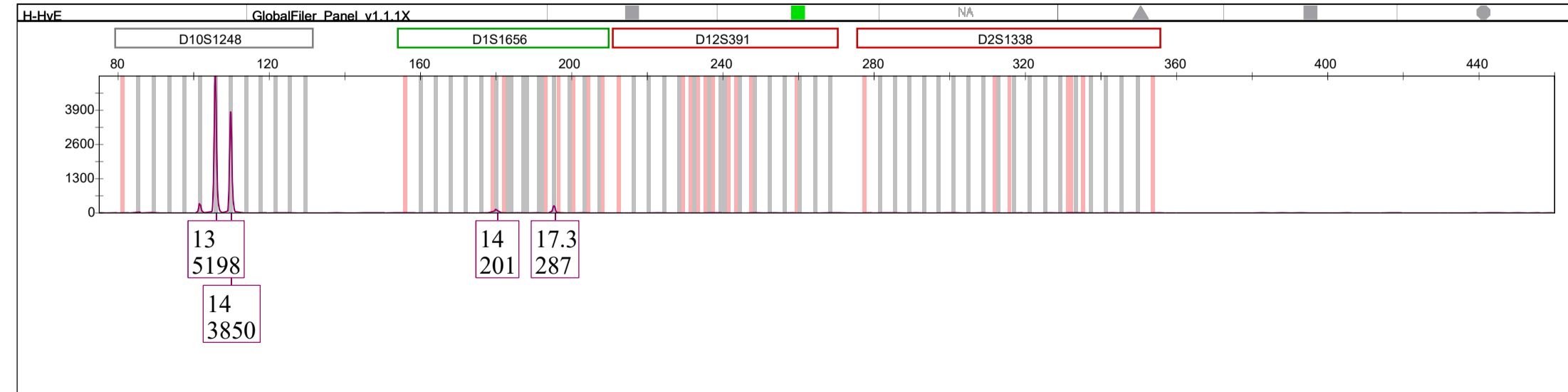

Supplement: Supplementary file 1 [file genes-16-01416-s001.zip › Figure S1. 1HE.pdf]

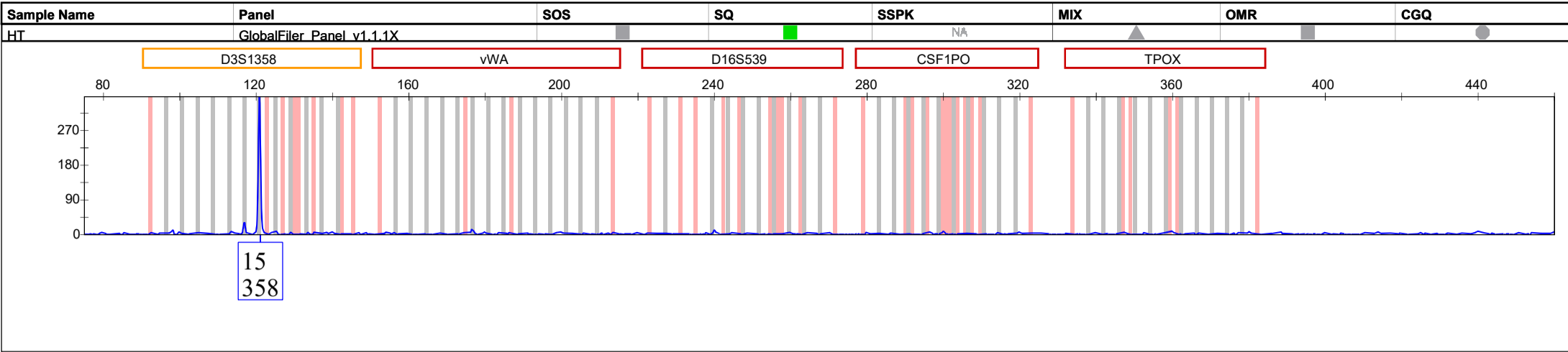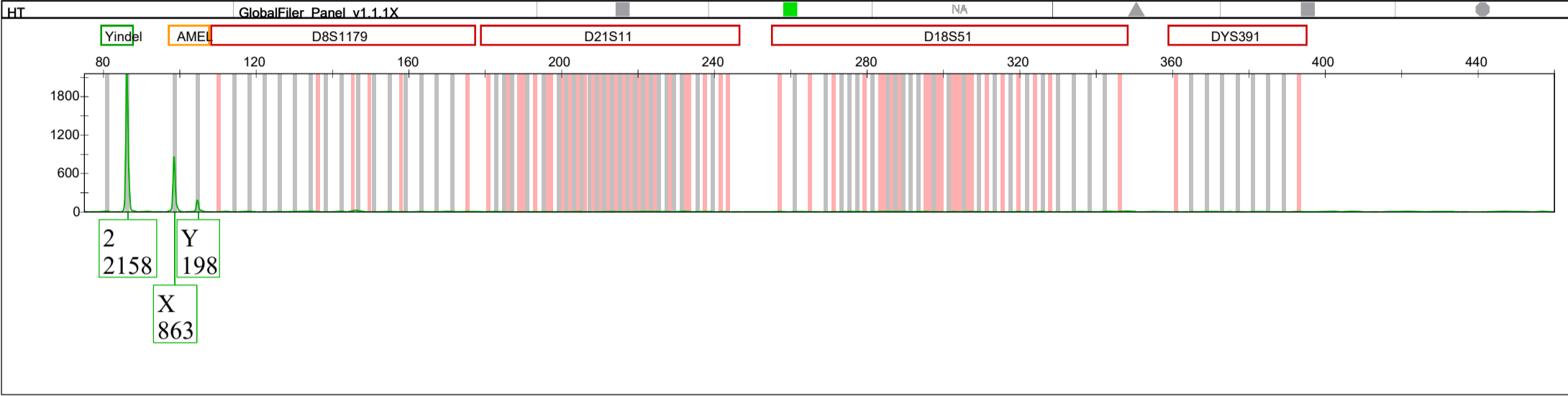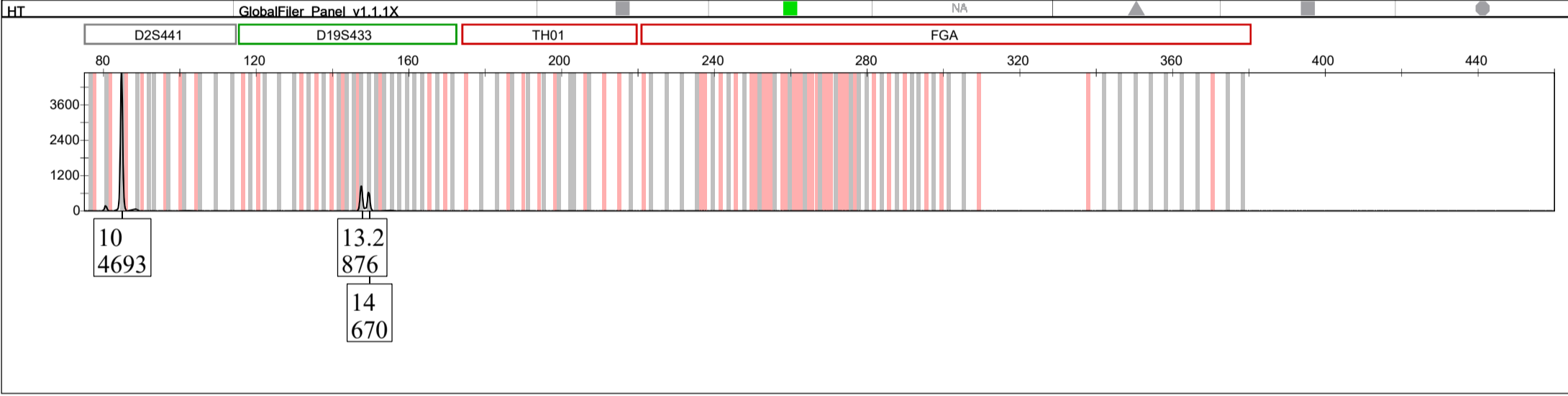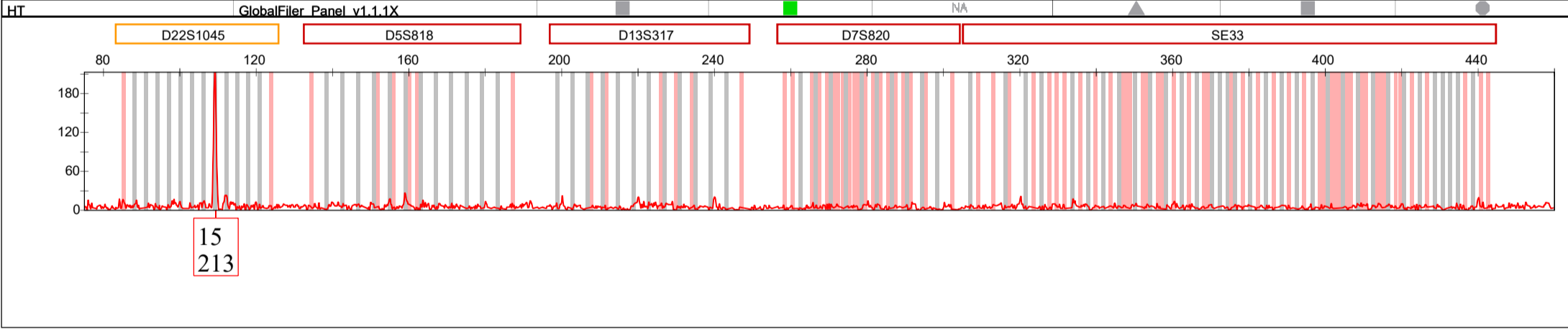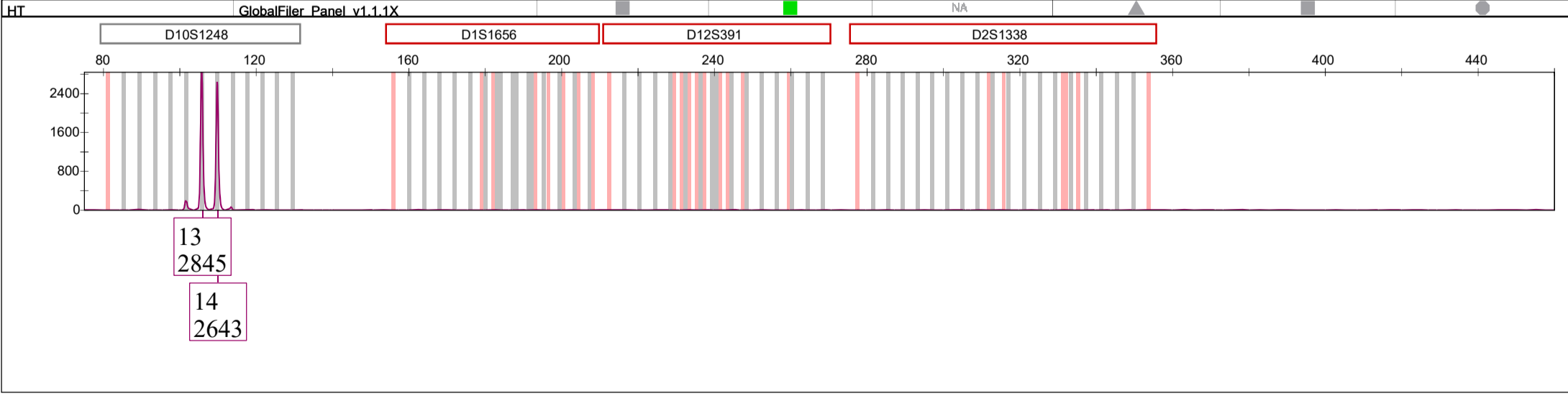

Supplement: Supplementary file 1 [file genes-16-01416-s001.zip › Figure S2. 1MT.pdf]

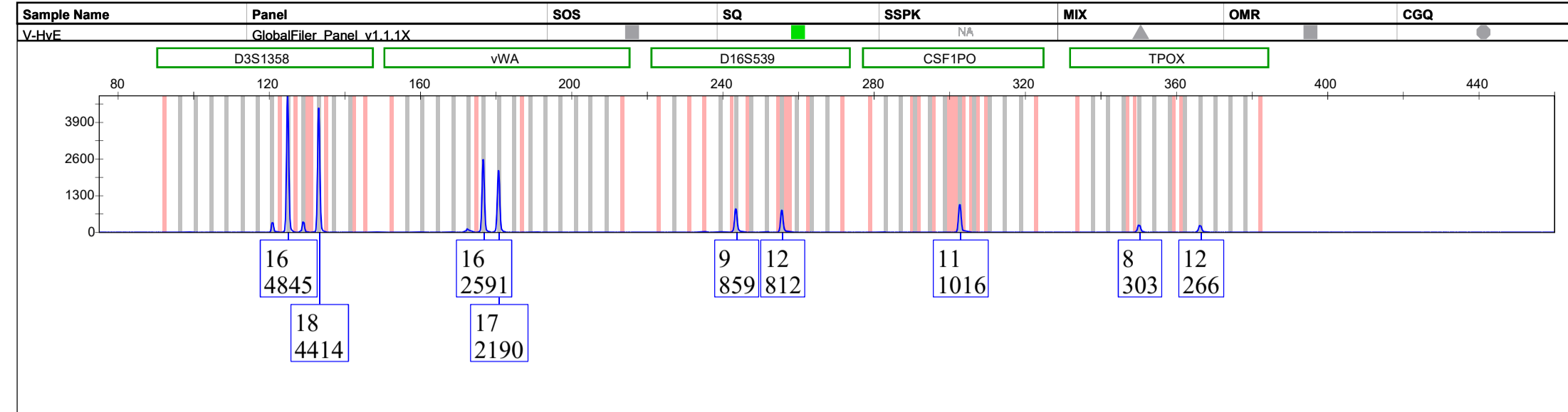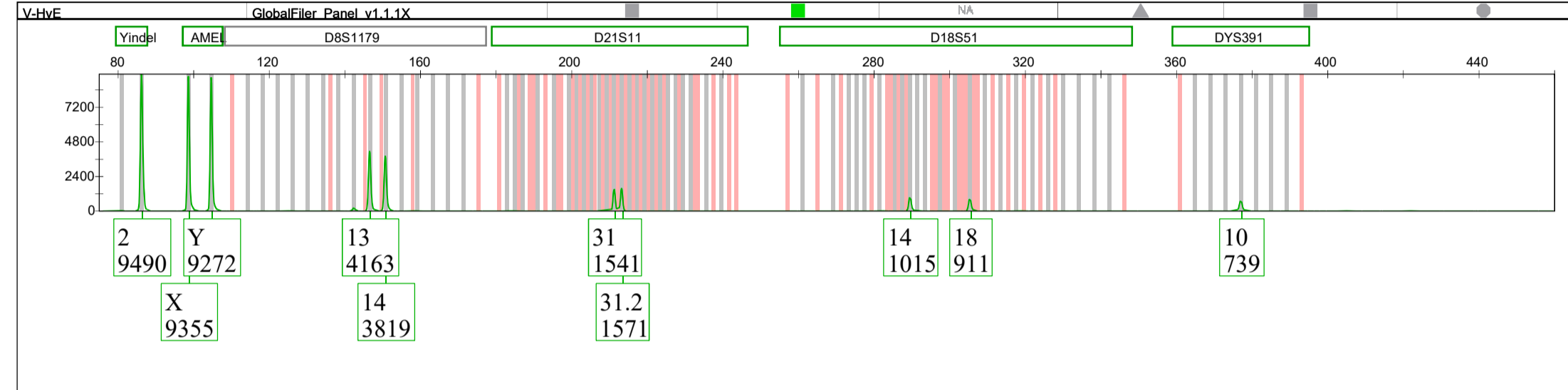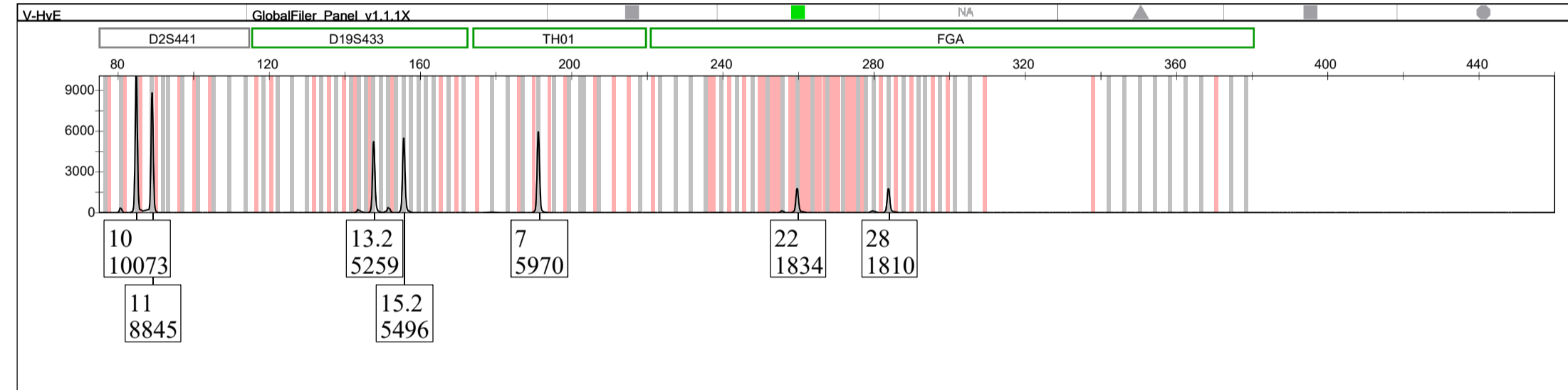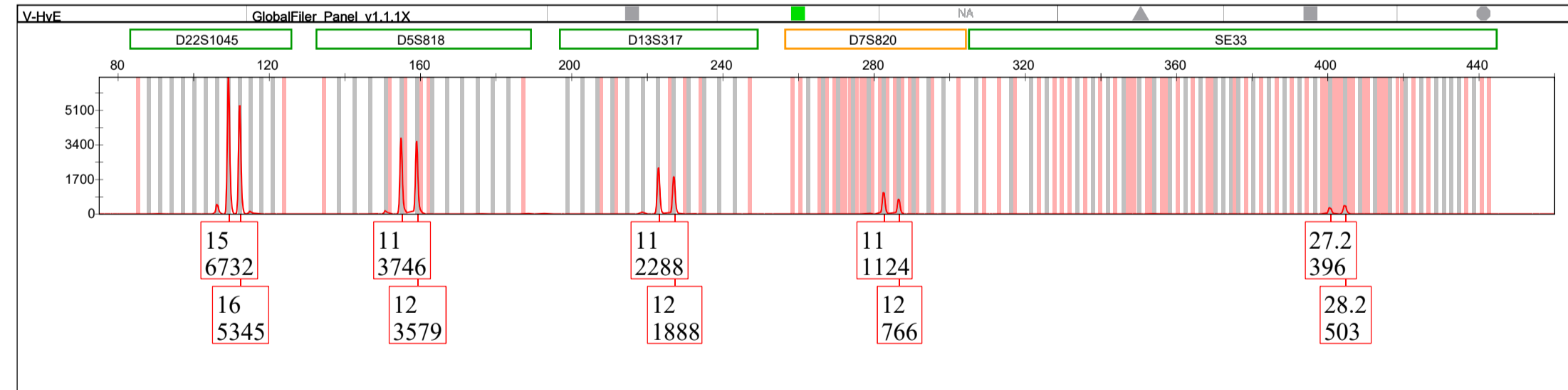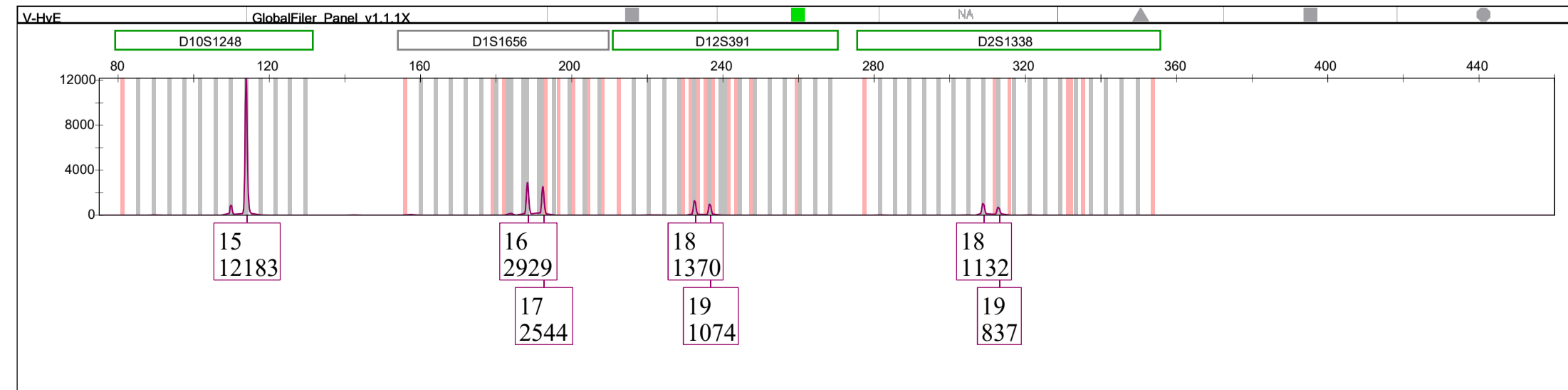

Supplement: Supplementary file 1 [file genes-16-01416-s001.zip › Figure S3. 2HE.pdf]

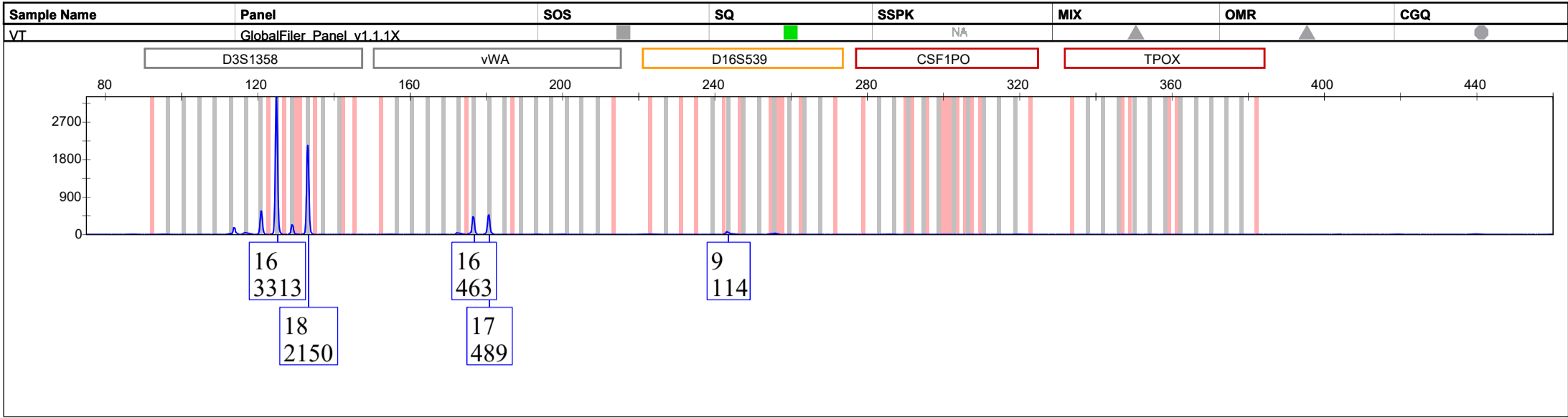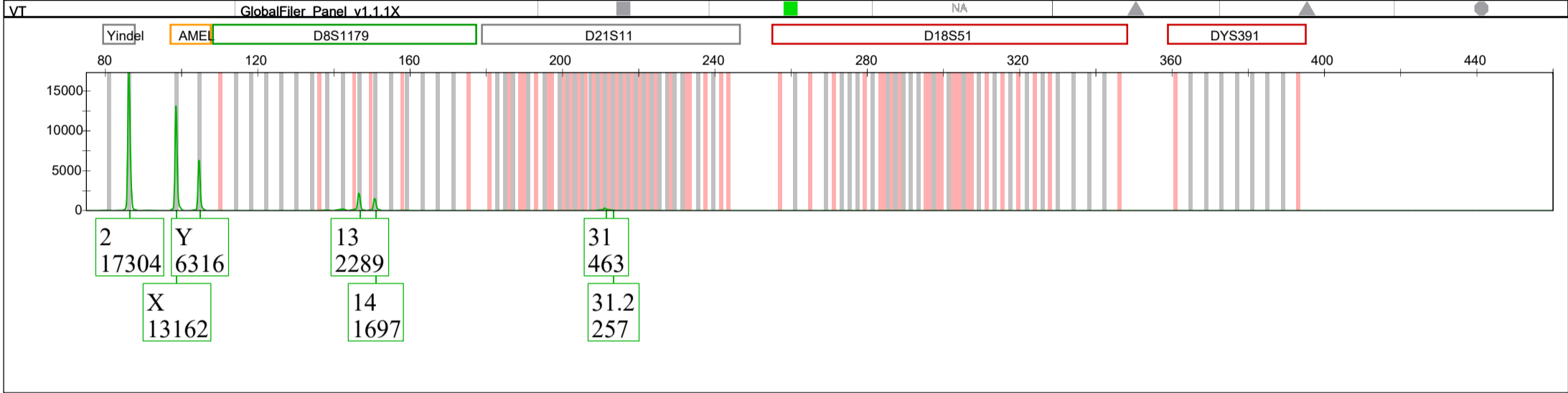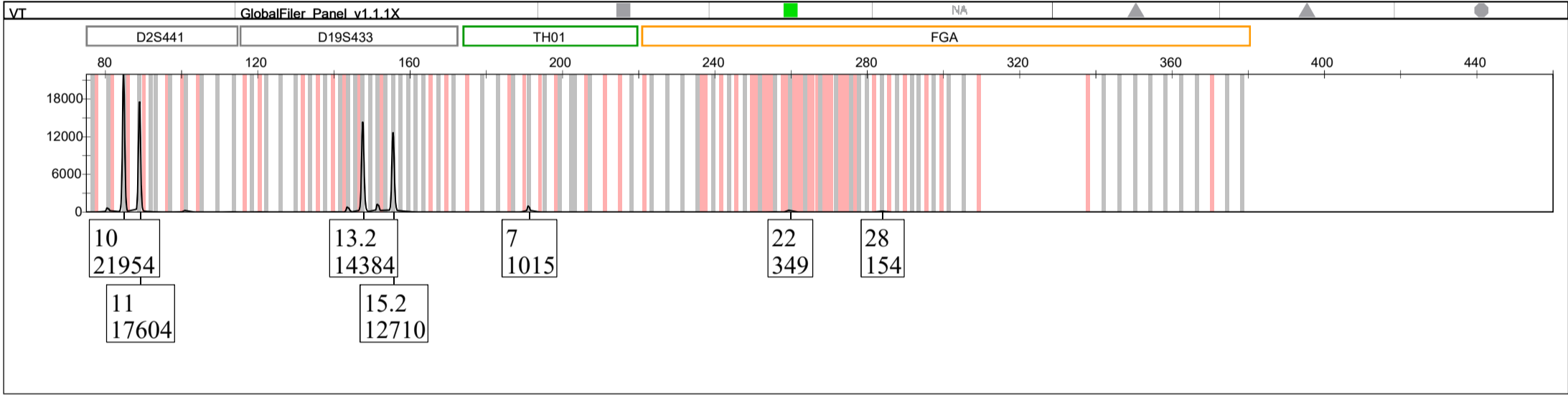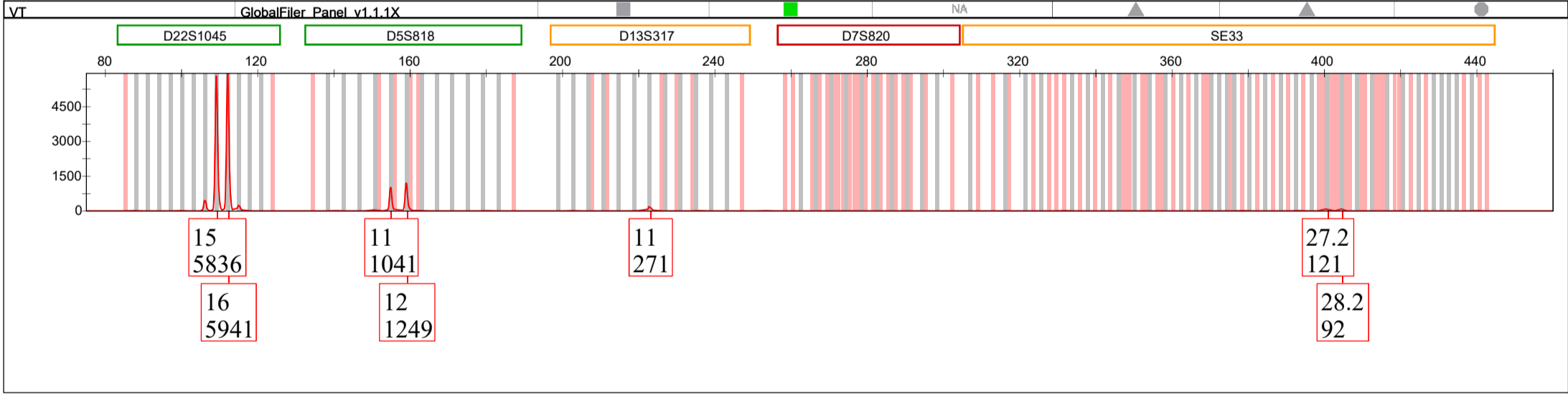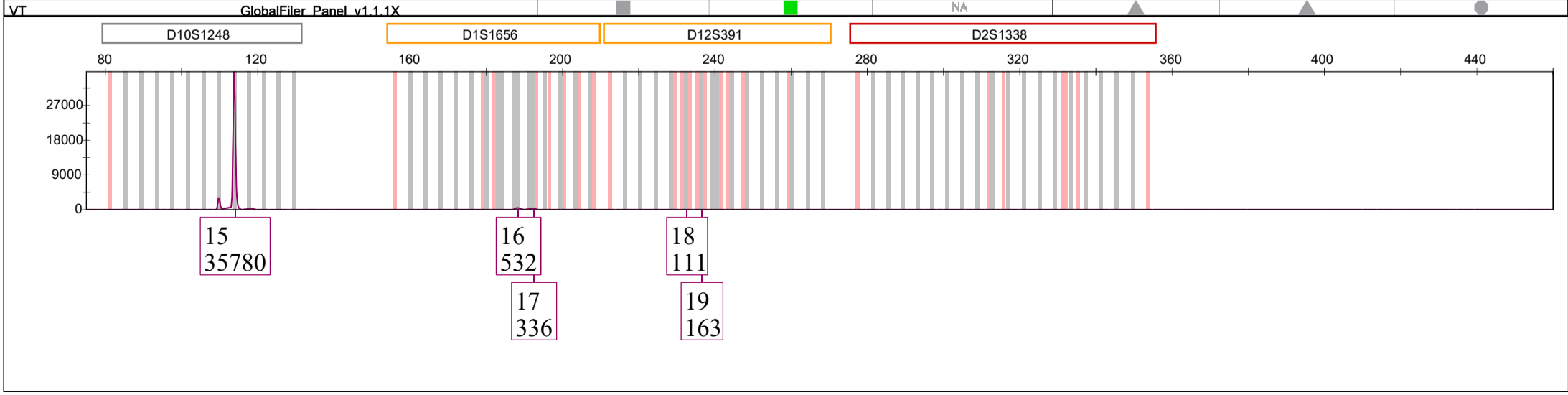

Supplement: Supplementary file 1 [file genes-16-01416-s001.zip › Figure S4. 2MT.pdf]

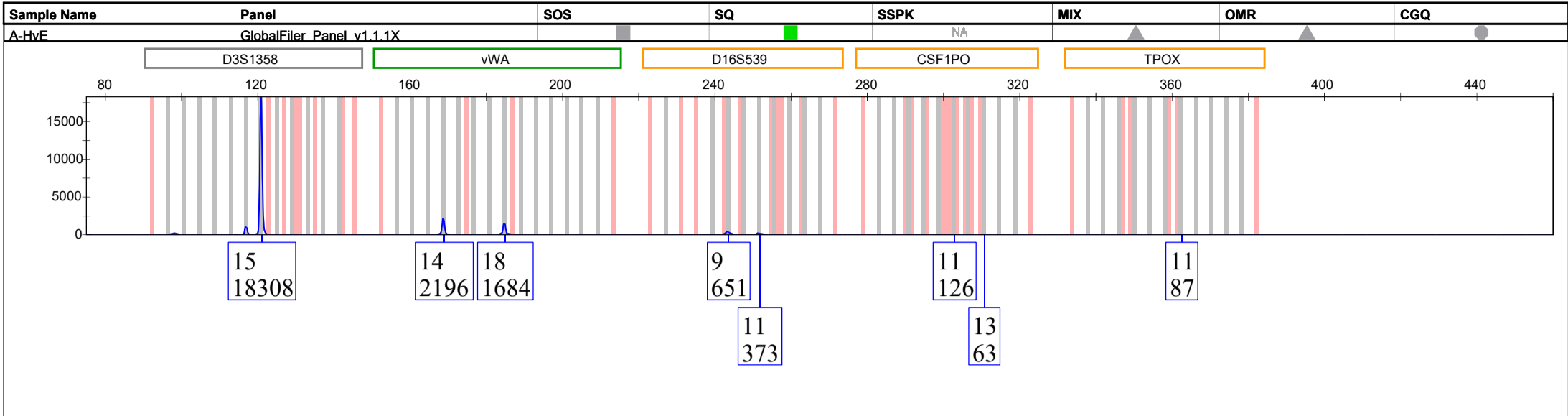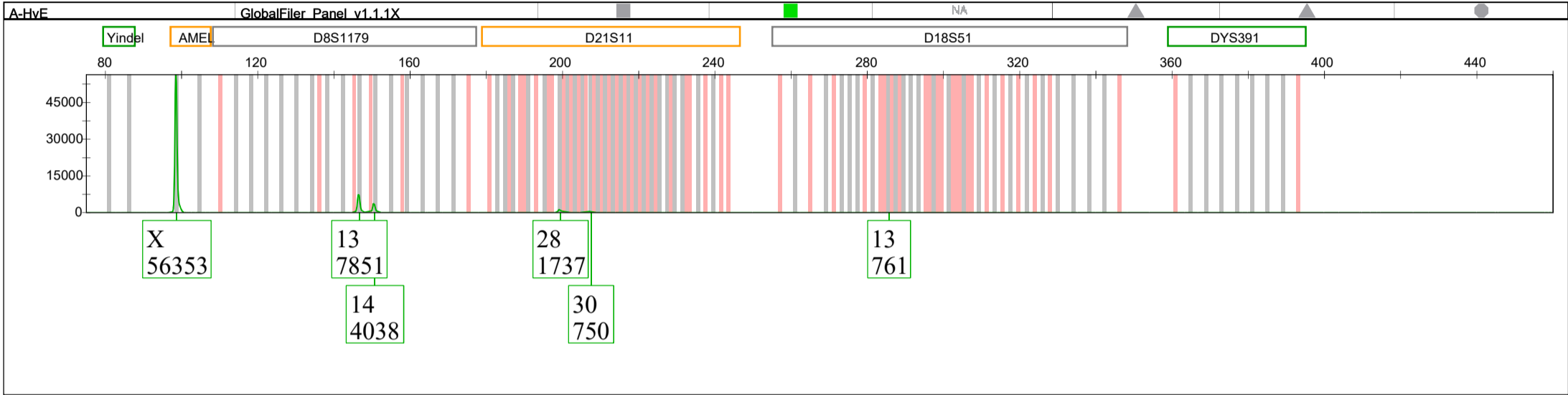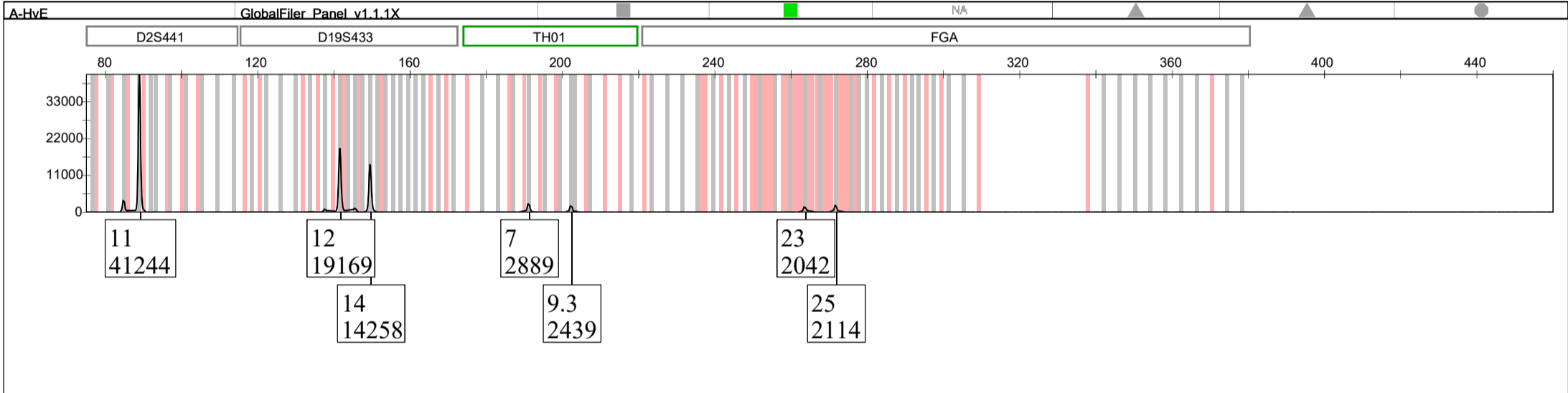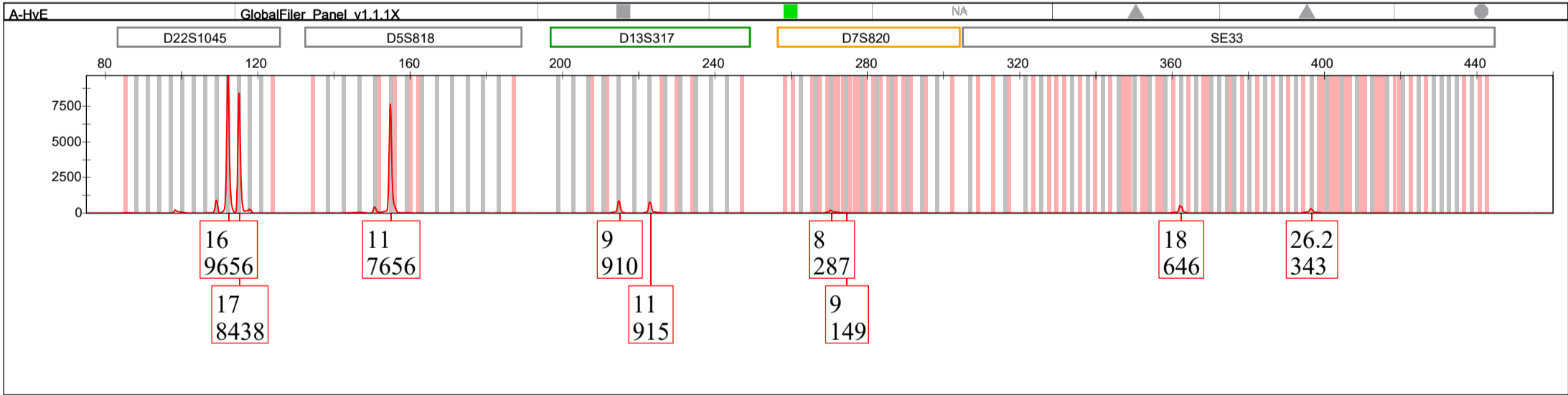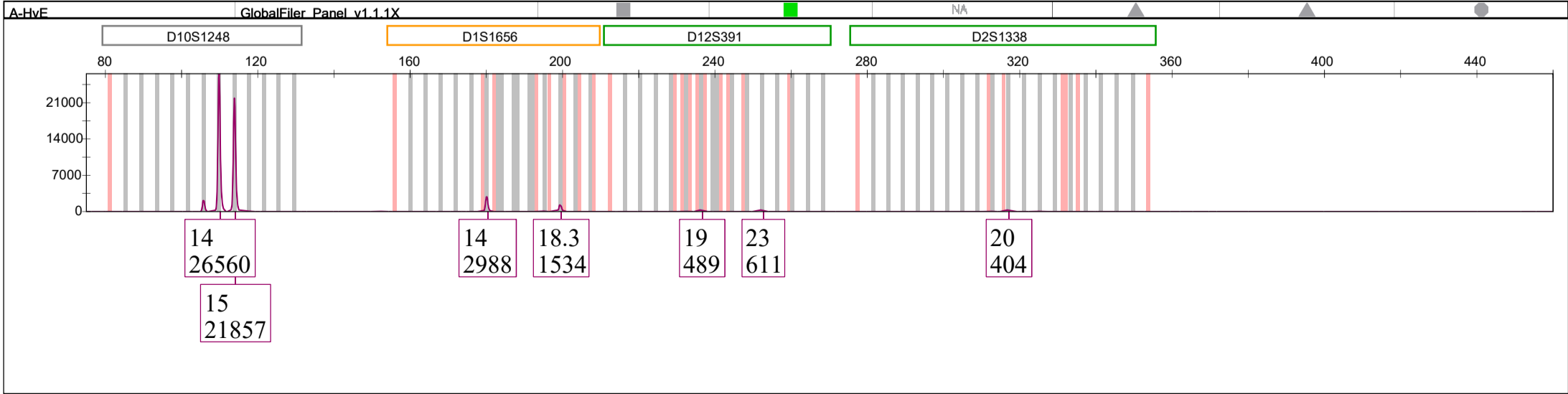

Supplement: Supplementary file 1 [file genes-16-01416-s001.zip › Figure S5. 3HE.pdf]

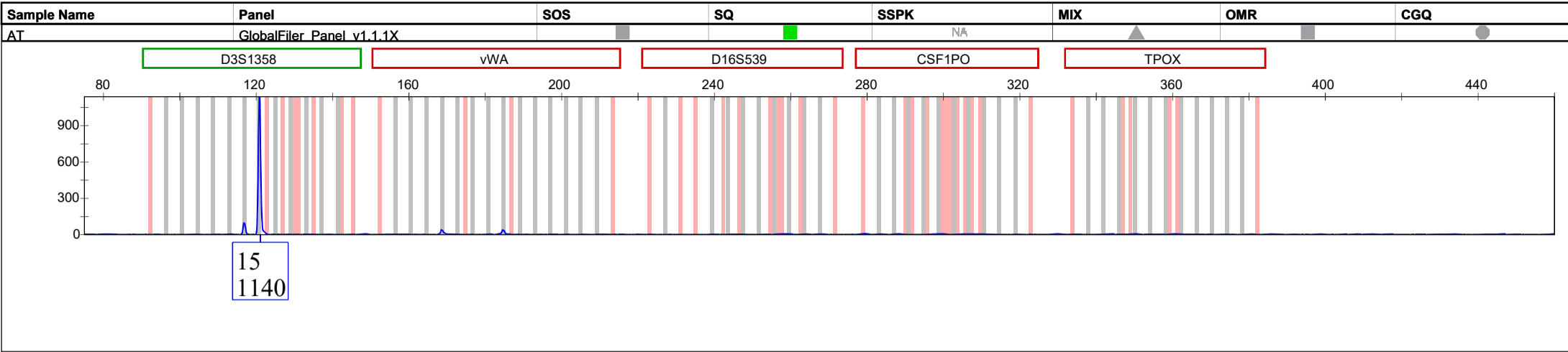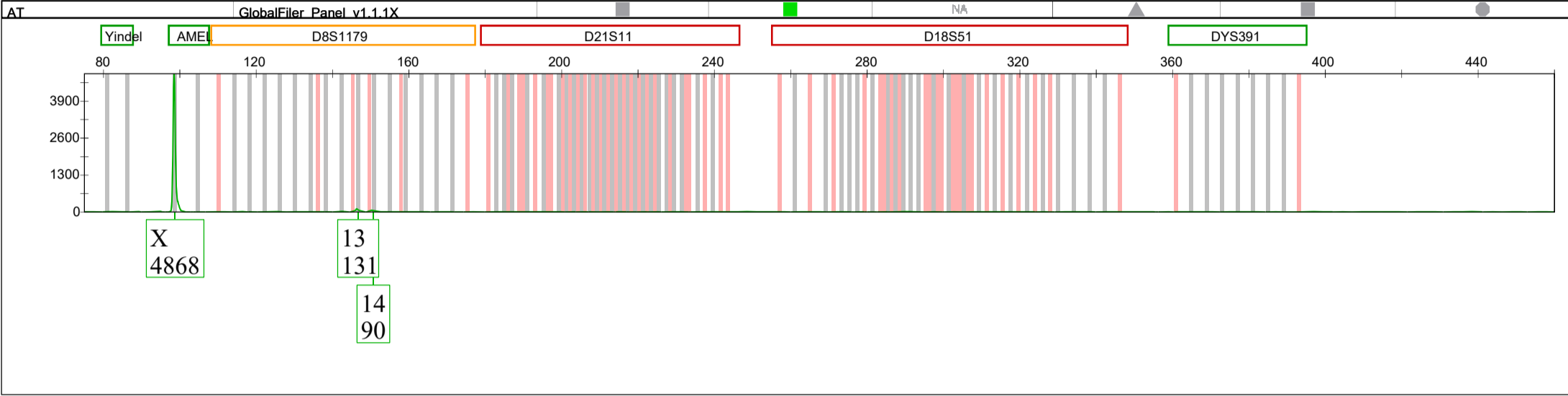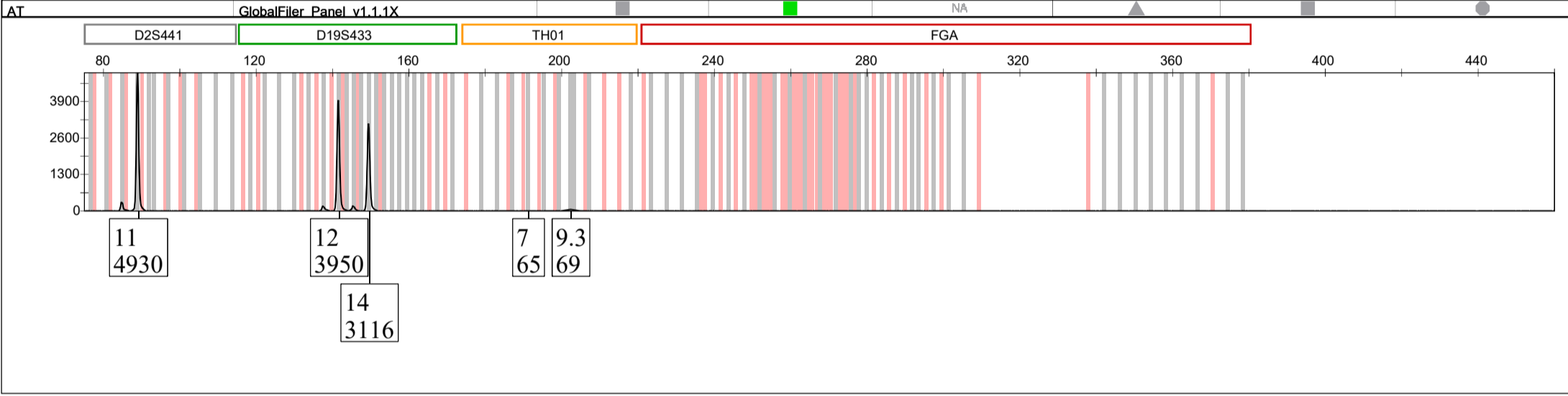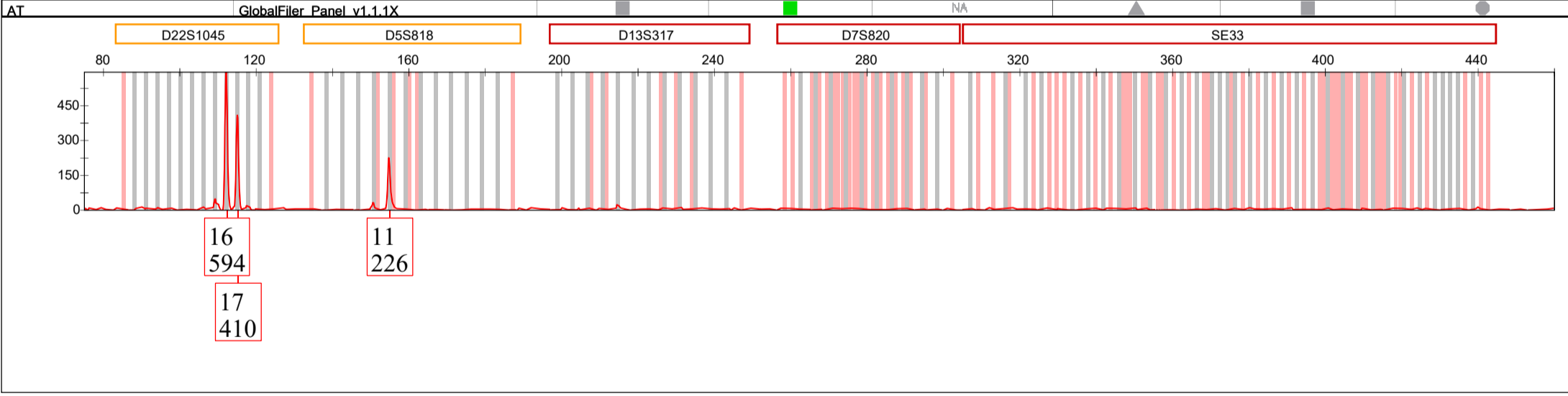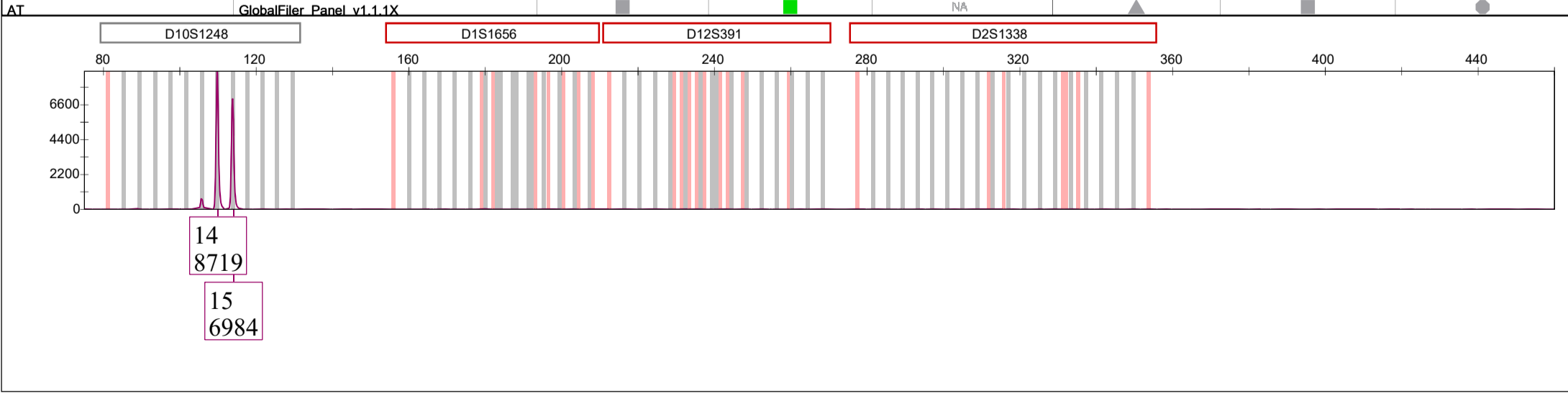

Supplement: Supplementary file 1 [file genes-16-01416-s001.zip › Figure S6. 3MT.pdf]
